# Case report: Preimplantation genetic testing for X-linked alport syndrome caused by variation in the *COL4A5* gene

**DOI:** 10.3389/fped.2023.1177019

**Published:** 2023-08-10

**Authors:** Nengqing Liu, Xiaojun Wen, Zhanhui Ou, Xiaowu Fang, Jing Du, Xiufeng Lin

**Affiliations:** ^1^ Reproductive Center, Zhongshan Boai Hospital, Zhongshan, China; ^2^ The Second Clinical College, Southern Medical University, Guangzhou, China

**Keywords:** X-Linked alport syndrome, *COL4A5*, next-generation sequencing, type IV collagen, preimplantation genetic testing

## Abstract

X-Linked Alport Syndrome (XLAS) is an X-linked, dominant, hereditary nephropathy mainly caused by mutations in the *COL4A5* gene, found on chromosome Xq22. In this study, we reported a pedigree with XLAS caused by a *COL4A5* mutation. This family gave birth to a boy with XLAS who developed hematuria and proteinuria at the age of 1 year. We used next-generation sequencing (NGS) to identify mutations in the proband and his parents and confirmed the results using Sanger sequencing. This testing showed there was a single nucleotide missense variation, c.3659G>A (p.Gly1220Asp) (NM_033380.3), in the *COL4A5* gene. To prevent the inheritance of the syndrome, we used eight embryos for trophoblast biopsy after assisted reproductive technology treatment, and whole genome amplification (WGA) was performed using multiple annealing and looping-based amplification cycles (MALBAC). Embryos were subjected to Preimplantation Genetic Testing (PGT) procedures, including Sanger sequencing, NGS-based single nucleotide polymorphism (SNP) haplotype linkage analysis, and chromosomal copy number variation (CNV) analysis. The results showed that three embryos (E1, E2, and E4) were free of CNV and genetic variation in the *COL4A5* gene. Embryo E1 (4AA) was transferred after consideration of the embryo growth rate, morphology, and PGT results. Prenatal diagnosis in the second trimester showed that the fetus had a normal karyotype and did not carry the *COL4A5* mutation (c.3659G>A). Ultimately, a healthy boy was born and did not carry the pathogenic *COL4A5* mutation, which indicated that PGT prevented the intergenerational transmission of the causative mutation of XLAS.

## 1. Introduction

Alport syndrome (AS) is the second rare hereditary renal failure syndrome, after autosomal dominant polycystic kidney disease, with an incidence of approximately 0.043% (1). The main clinical features are hematuria, proteinuria, progressive renal failure, end-stage renal disease, hearing loss, and eye abnormalities. The syndrome occurs via three inheritance modes: X-linked dominant inheritance, autosomal dominant inheritance, and autosomal recessive inheritance (2). The X-linked dominant version of AS (XLAS) involves mutation of the *COL4A5* gene and is the most common type, accounting for more than 80% of cases (3). The *COL4A5* gene is located on chromosome Xq22.3 and has a total length of 277.7 kb, with 53 exons (NM_033380.2). It encodes the type IV collagen α5 [α5(IV)] chain, which is the main component of the glomerular basement membrane (GBM), cochlea, and critical structural components of the eye. The ClinVar database details more than 1,800 variants of *COL4A5*, including deletions, frameshift mutations, nonsense mutations, splicing, and missense mutations. However, 70% of the variants are single nucleotide missense mutations. Mutations in the *COL4A5* gene may disrupt the triple-helix folding and reduce the secretion of type IV collagen, ultimately resulting in defects in the basement membrane (4). There are significant sex differences in the clinical manifestations of XLAS patients, and male patients have more severe course and symptoms. Male patients with XLAS exhibit proteinuria and hematuria in early childhood, and more than 90% develop end-stage renal failure before the age of 40, with a median age of 25 years. Some patients may occur varying degrees of hearing loss and ocular changes (5). Male patients also have a solid genotype-phenotype association (6, 7), with truncating variants causing the most severe phenotype and the youngest age for the development of ESRD. Splicing variants have a moderate phenotype, and missense variants have a mild phenotype. Female XLAS patients have more extensive and milder clinical manifestations, mainly hematuria. There is a lack of apparent genotype-phenotype association, and only 15% of women will develop ESRD by age 60 (8–10).

People with XLAS face a significant lifetime risk of kidney failure, sensorineural deafness, and ocular abnormalities (11). Effective intervention for XLAS depends upon early diagnosis, and blocking the spread of the *COL4A5* mutation is a fundamental solution. Preimplantation genetic testing (PGT) is defined as a type of assisted reproductive technology that is used to analyze the DNA of oocytes (polar bodies) or embryos (cleavage stage or blastocyst) to confirm genetic abnormalities (12). Preimplantation genetic testing is designed to block intergenerational transmission of disease-causing variants to avoid the need for therapeutic termination of pregnancy. The testing includes PGT for aneuploidy (PGT-A), PGT for chromosomal structural rearrangement (PGT-SR), and PGT for single genes/single gene defects (PGT-M) (13). Here, we reported an XLAS family in which a c.3659G>A (p.Gly1220Asp) mutation in the *COL4A5* gene was identified in the wife and child. The couple underwent genetic counselling and PGT-M to have a healthy child. Based on multiple annealing and looping-based amplification cycles (MALBAC), next-generation sequencing (NGS) technology, and single nucleotide polymorphism (SNP) haplotype analysis, we were able to help the couple to have a healthy child. That proved the PGT-M was a viable way to block the inheritance of XLAS by the next generation.

## 2. Methods

### 2.1. Subjects and ethics approval

In 2017, a couple had a boy admitted to the Department of Pediatrics, Zhongshan Bo'ai Hospital when he was one year old due to hematuria and proteinuria. Alport syndrome (AS) was suspected from the clinical manifestations and renal pathology biopsy results. In 2020, the boy underwent genetic testing, and the results showed that he had an X-linked hemizygous *COL4A5* mutation, c.3659G>A (p.Gly1220Asp) (NM_033380.3). The couple visited the Zhongshan Bo'ai Hospital Reproductive Centre for genetic and fertility counselling to have another child. Clinical evaluation, sample collection, pedigree analysis, PGT, and assisted reproductive technology (ART) were performed at the Reproductive Center of Zhongshan Bo'ai Hospital. This study was approved by the internal ethics committee of Zhongshan Bo'ai Hospital, and informed consent was obtained from the couple.

### 2.2. Genomic DNA extraction and variant site detection

Genomic DNA (gDNA) was isolated from the couple's and their children's peripheral blood using the TIANamp Blood DNA kit (Tiangen Biotechnology, Cat# DP348-02). Polymerase Chain Reaction (PCR) amplification and Sanger sequencing were conducted to validate the mutation sites in *COL4A5*. The forward (GGGTAGATTTGGGATTTGGT) and reverse (CTACCACTCACGGACATACC) primers (synthesized by Guangzhou IGE Biotechnology) were designed using Primer 5.0 software to cover the coding exons and flanking introns of *COL4A5*, for PCR amplification of the mutation. A VeritiPro™ 96-well thermal cycler (Thermo Fisher Scientific, Cat# 7394) was used for Quantitative-PCR (Q-PCR) analysis with SYBR® Premix Ex Taq™ II (Takara Biotechnology, Cat# RR820A). Sanger sequencing data were analyzed by ChromasPro software (Yikon Genomics). Database and literature surveys were conducted on the mutation sites. The type IV collagen α5 chain and type IV collagen 3D protein structures were assembled using PyMOL software.

### 2.3. Identification of pathogenic mutations

Paired-end sequencing library preparation was performed on the genomic DNA of the proband using a DNA sample preparation kit (NEBNext). The amplified DNA was captured using a GenCap capture kit. The enrichment libraries were sequenced on an Illumina HiSeq X ten sequencer for paired reads of 150 bp. After sequencing, the raw data were saved in FASTQ format for bioinformatics analysis as follows: Firstly, Illumina sequencing adapters and low-quality reads (<80 bp) were filtered by Cutadapt. After quality control, the clean reads were mapped to the UCSC hg19 human reference genome using BWA. Duplicated reads were removed using Picard tools, and mapping reads were used for variation detection. Secondly, the SNP and InDel variants were detected using GATK HaplotypeCaller, and GATK VariantFiltration was used to filter the variants. Five steps were used to select the potential pathogenic mutations in the downstream analysis: (I) there should be more than five mutation reads, and the mutation ratio should be no less than 30%; (II) mutations were removed if their frequency was more than 5% in 1,000 g, ESP6500, and our in-house database; (III) mutations were dropped if they existed in the InNormal database (MyGenostics); (IV) synonymous mutations were removed; (V) after I, II and III, if the mutations were synonymous and they were reported in HGMD, they were not removed. After these five steps, the remaining mutations should be pathogenic.

### 2.4. Assisted reproductive technology procedure and embryo biopsy

Ovarian stimulation was performed using the standard long protocol of gonadotropin-releasing hormone (GnRH) agonist administration (long GnRH agonist protocol). At day 20 + 1 of the menstrual cycle, a GnRH agonist was used for initial down-regulation. Gonadotropins (mostly recFSH or HMG) were administered on day two of the subsequent cycle to promote adequate follicle development. Ovulation was triggered by HCG injection when follicles reached 18 mm in diameter. Oocytes were then extracted after 36 h using an ultrasound-guided follicular puncture. Sperm samples were prepared using the density gradient technique, and swim-up sperm were selected for intracytoplasmic sperm injection (ICSI). In accordance with program standards, embryos were produced using ICSI and cultivated up until the blastocyst stage. We observed the formation and morphology of blastocysts on day five and day six and scored them using Gardner's scoring system. The blastocysts with a Gardner score of 3BB or higher were considered suitable for embryo biopsy. The blastocyst trophoblasts were cut with a laser to obtain four to six trophectoderm (TE) cells for PGT-M.

### 2.5. Whole genome amplification

We transferred TE cells to a 0.2 µl PCR tube that contained 4.5 µl of lysis buffer (Yikon Genomics, Suzhou, China) for whole genome amplification (WGA). The WGA of each embryo biopsy sample was performed using a MALBAC WGA kit (Yikon Genomics, XK-028-24, Suzhou, China), following the manufacturer's instructions. The collected cells were initially lysed in a lysis buffer, followed by MALBAC pre-amplification and exponential amplification, to obtain 2–5 μg of DNA.

### 2.6. Next-generation sequencing, copy number variation analysis, and single nucleotide polymorphism haplotype analysis

The WGA product was used as the template for library preparation. The NGS library building kit (Yikon Genomics, KT100804248, Suzhou, China) performed enzyme digestion, terminal repair, joint connection, DNA purification, PCR enrichment, and PCR product purification. Libraries were sequenced on the MiSeq Dx platform (Illumina, California, United States). Raw data was automatically filtered to generate a FASTQ file in which the Q30 value was greater than 90%. The CNV analysis of the FASTQ files was performed to report any deletions or duplications of more than 4 Mb for each embryo. Valid reads of more than 1 Mb and CV (1,000 K_bin_size) were considered to be within the acceptable range. High-frequency SNP sites in the 2 Mb regions upstream and downstream of the pathogenic gene and within the gene were screened as genetic markers. These sites were submitted to the https://www.ampliseq.com/ website to design a primer pool. Using WGA products and gDNA as templates, SNP library preparation was carried out under the instructions for the NGS library preparation general kit (Yikon Genomics, KT100804424, Suzhou, China). That included multiple specific amplification of upstream and downstream SNP panels of mutant genes, purification of amplification products, enzymatic hydrolysis of non-target fragments, purification of enzymatic hydrolysis products, PCR enrichment library construction, PCR product purification and the establishment of linkage relationships by family pedigrees, to diagnose embryo pathogenicity. After creating the SNP library, a Nextseq550 sequencer (Illumina, California, United States) was used to complete the sequencing. CNV and SNP data were analyzed using ChromGo bioinformatics analysis software (Yikon Genomics, Suzhou, China).

## 3. Results

### 3.1. Patients and relatives

**Figure 1:**
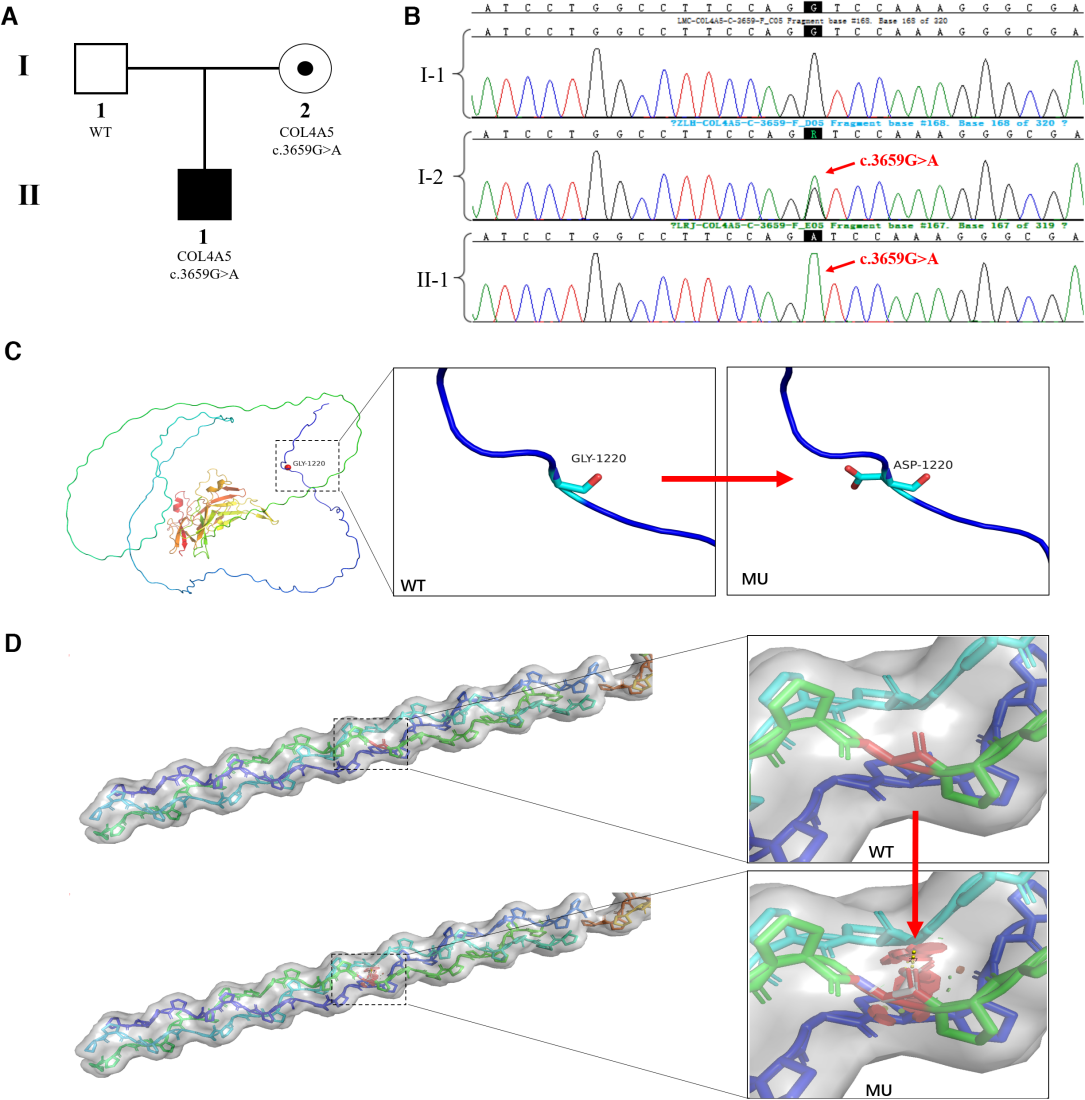
*COL4A5* mutations and identification of affected families. (**A**) Pedigree of an XLAS-affected family: The proband (II-1), a 1-year-old boy, was identified as having XLAS caused by a pathogenic *COL4A5* mutation; the mother (I-2) was also identified as having the same mutation but no clinical manifestations; the father was normal. (**B**) Sanger sequencing confirmed the hemizygous mutation of *COL4A5* (c.3659G>A (p.Gly1220Asp) (NM_033380.3) in the proband (II-1) and his mother (I-2); the father did not have any mutations in the *COL4A5* gene. (**C**) The crystal structure of the α5 chain of type IV collagen. The glycine at position 1,220 in the protein's primary structure is replaced by aspartic acid when the c.3659G>A mutation occurs. (**D**) Steric hindrance is introduced into the tertiary structure of type IV collagen due to the c.3659G>A (p.Gly1220Asp) mutation.

When the child was one year old, he developed gross hematuria for no apparent reason. His body temperature, respiration, and blood pressure were average. There was no oedema of the face and lower limbs, no percussion pain in the kidney area, and no other abnormalities found during the physical examination. Urine testing showed 3+ occult blood and 1+ urine protein. The 24 h urine protein quantification was 213.46 mg/kg, the urine protein/creatinine ratio was 38.21 mg/g, and other liver and kidney functions, myocardial enzymes, electrolytes, and complements were normal. Colour Doppler ultrasonography showed no obvious abnormalities in the kidneys, bladder, and renal blood vessels. Urine microbial cultures were negative. Hearing and fundus examinations showed no abnormalities. A pathological kidney biopsy showed negative tissue immunofluorescence staining of α3 and α5 (IV) in the basement membranes, which suggested α3 and α5 chain deletion, and a basement membrane diameter of 180 nm. AS was clinically suspected. The boy's father was asymptomatic, and his mother only showed intermittent isolated hematuria. Peripheral blood samples from the proband and his parents were collected for pedigree analysis. The NGS results showed that the proband and his mother had the c.3659G>A (p.Gly1220Asp) (NM_033380.3) mutation in the *COL4A5* gene, but this was not identified in his father (Figures 1A,B). We used the American College of Medical Genetics and Genomics (ACMG/AMP) Sequence Variation Interpretation Standards and Guidelines to classify the mutation as follows.

The c.3659G>A mutation in the *COL4A5* gene was absent from population databases (PM2).

The region where the mutation was located was an essential part of this protein, and the amino acid sequence of different species was highly conserved. The mutation interrupted the Gly-X-Y domain of the collagen gene (PM1) (Figures 1C,D).

Computational evidence was used to predict the damaging effect of the mutation on the protein (PP3). The p.Gly1220Asp mutated protein has been found in AS patients (14) (PP4).

The mutation complied with the interpretation rules of PM2, PM1, PP3, and PP4 and was classified as “Category 2- Likely Pathogenic”.

This mutation inherited in an X-linked dominant manner was the cause of XLAS. The karyotype analysis of the couple showed normal karyotypes.

### 3.2. Blastocyst culture, trophoblast biopsy, and sanger sequencing

**Figure 2:**
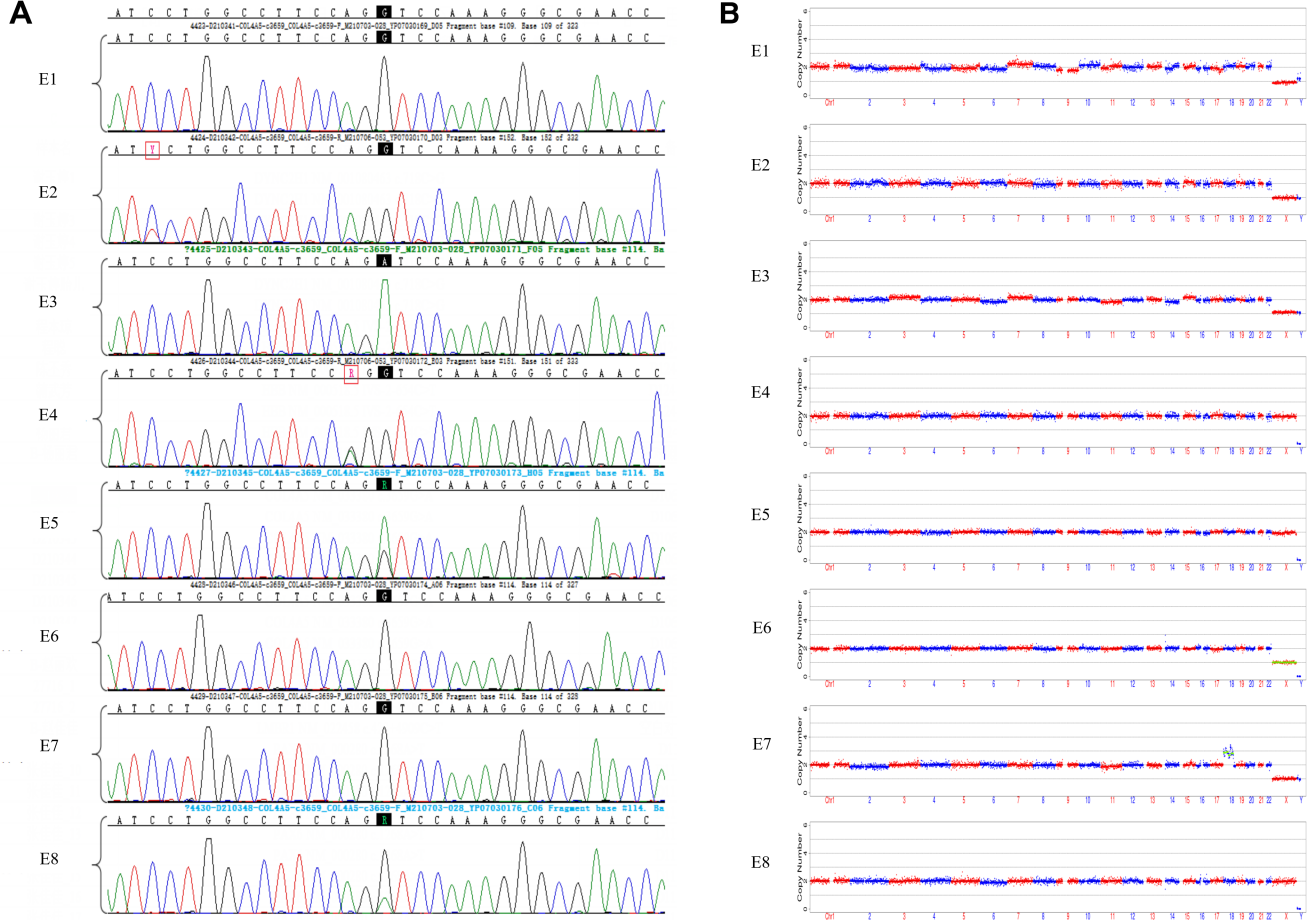
Results of sanger sequencing and CNV analysis of WGA products from embryonic trophoblast biopsy cells. (**A**) Sanger sequencing chromatograms of eight embryos. Mutation sites are shown with a black background. The E1, E2, E4, E6, and E7 embryos did not inherit the *COL4A5* pathogenic mutation (c.3659G>A), while E3, E5, and E8 were found to have the mutation; (**B**) CNVs in eight embryos, at low NGS sequencing depth. NGS analysis of embryos E1, E2, E3, E4, E5, and E8 did not reveal any large CNVs or copy number abnormalities. Both E6 and E7 had copy number abnormalities.

We obtained 20 oocytes, under ultrasound guidance, after ovulation induction. These metaphase II (MII) oocytes were subsequently inseminated by ICSI, and all were fertilized normally, as indicated by the presence of two pronuclei. Eight blastocysts were obtained on the fifth day after insemination and scored by the Gardner Grade (Table 1). All blastocysts underwent trophectoderm biopsy on day 6 to collect four to six cells. After cell lysis, WGA was performed using the MALBAC two-step method, and the products were purified. The purified products were detected by amplification of target fragments and Sanger sequencing. The results showed that the c.3659G>A mutation was present in the *COL4A5* gene in embryos E3, E5, and E8, while no mutation was found in E1, E2, E4, E6, or E7 (Figure 2A).

**Table 1:** Summary of detection results.

Embryo number	Biopsy time	Gardner grade	Copy number variations	SNP haplotype	Sanger sequencing
E1	D6	4AA	46, XY	Normal male	Normal
E2	D6	4AA	46, XY	Normal male	Normal
E3	D6	4AA	46, XY	Abnormal detection	c.3659G>A
E4	D6	4AA	46, XX	Normal female	Normal
E5	D6	4AB	46, XX	Abnormal detection	c.3659G>A
E6	D6	4BB	45, X, -X(×1)	Maternal haplotype	Normal
E7	D6	3AA	46, XY, +18p(×3), +18q(q11.2 → q12.1, ∼12 Mb, ×3), +18q(q12.1 → q12.3, ∼12 Mb, ×3, mos, ∼69%), +18q(q12.3 → q21.1, ∼5 Mb, ×3), +18q(q21.1 → q22.1, ∼16 Mb, ×3)	Abnormal detection	Normal
E8	D6	3BB	46, XX	Normal female	c.3659G>A

### 3.3. Preimplantation genetic testing for aneuploidy (PGT-A)

The NGS-based CNV sequencing of WGA purified products on the Illumina MiseqDx platform was used to detect large CNVs of more than 4 Mb and 30%–70% mosaicism (larger than 10 Mb). No CNVs larger than 4 Mb or aneuploidy were found in embryos E1, E2, E3, E4, E5, or E8. However, E6 had a monosomy of the X chromosome, and E7 was aneuploid (Figure 2B).

### 3.4. SNP haplotype analysis

**Figure 3:**
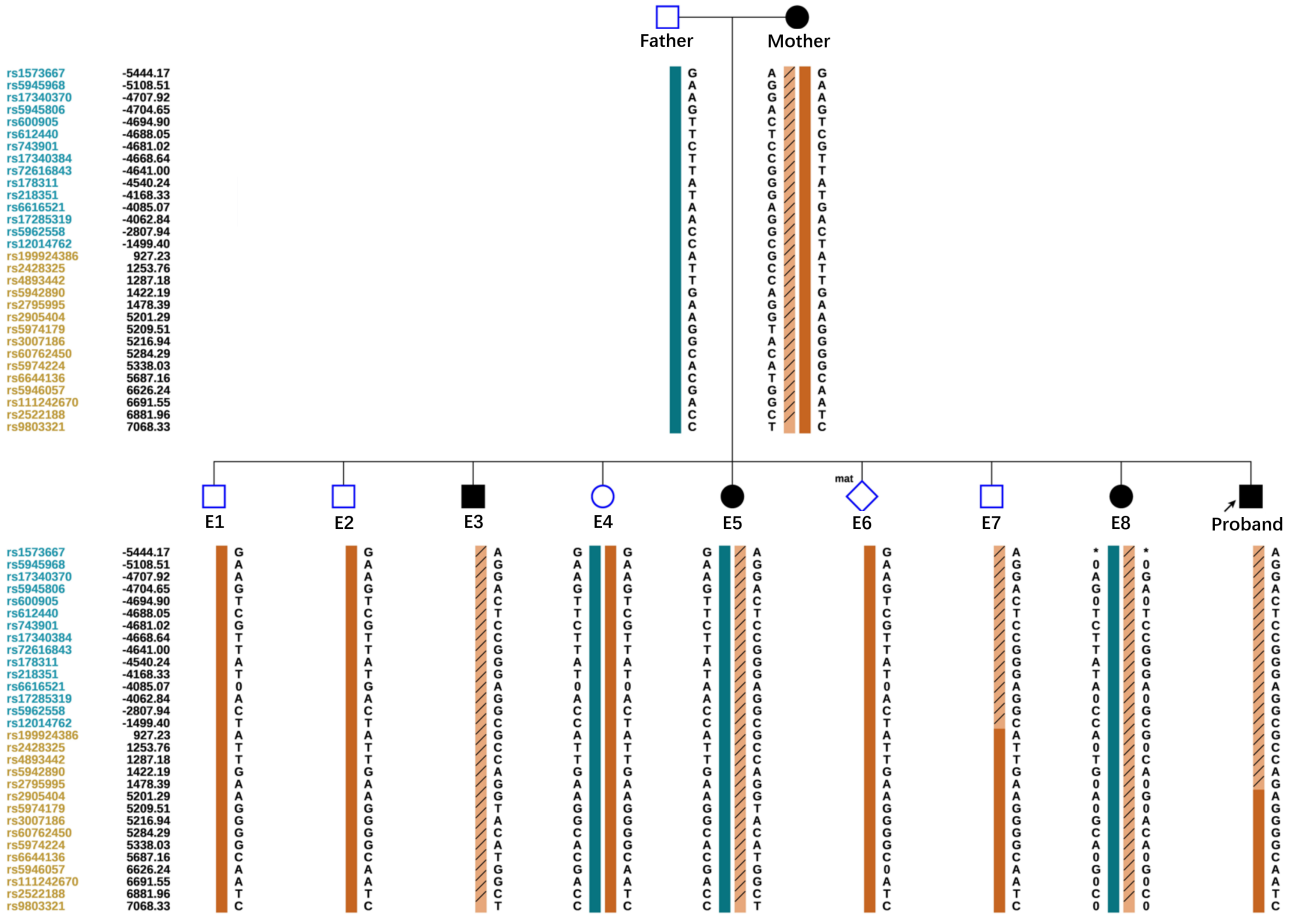
Schematic representation of the SNP-based haplotypes of the family members and embryos. The SNP sites are indicated on the left, and blue and orange represent SNP sites upstream or downstream, respectively, of the chromosomal position where the gene is located. Light orange left slashes to represent high-risk haplotypes, dark blue bars represent normal or low-risk haplotypes of the father, and dark orange bars represent normal or low-risk haplotypes of the mother. The results showed that E1, E2, E4, and E7 were normal embryos. E6 had the maternal haplotype.

Allelic dropout (ADO) may occur during gene amplification. In this disease, ADO may cause diseased embryos to be misdiagnosed as normal embryos. To reduce ADO interference on diagnostic results, the peripheral blood gDNA of the couple and their child (proband), plus the WGA products of the embryos, were analyzed for SNP haplotypes (Figure 3). We performed linkage analysis using the SNP markers within the 2 Mb region that flanked the *COL4A5* gene. Sixty informative polymorphic SNPs were selected to establish haplotypes. In this case, 15 SNPs within 1 Mb upstream and downstream of the mutation site were used for the analysis. When combined with family haplotypes (Figure 3), the results showed that embryos E1, E2, E4, E6, and E7 did not carry the maternal mutation, although E6 had the maternal haplotype. Based on pathogenic mutation detection results and linkage analysis, E1, E2, E4, and E7 did not carry the *COL4A5* c.3659G>A mutation (Table 1).

### 3.5. Embryo transfer and prenatal genetic diagnosis

The couple had discussions with a reproductive doctor, embryologist, and medical geneticist to determine the order of embryo transfer at our Center. Based on the developmental stage grade of the embryos, the results of the Sanger sequencing, CNV analysis, and SNP haplotype analysis, the final decision was made to transfer embryo E1 by consensus. A successful pregnancy was achieved (Table 1). At 20 weeks of gestation, the pregnant woman underwent ultrasound-guided amniocentesis, and amniotic fluid cells were cultured. Amniotic fluid cells were used for CMA, target fragment amplification, and Sanger sequencing. The results showed no abnormalities, and Sanger sequencing results were consistent with the PGT. The woman eventually gave birth to a healthy boy who did not carry the mutation.

## 4. Discussion

In this study, we reported an XLAS family with a mutation in the *COL4A5* gene on the X chromosome. The results of genetic analyses showed that there was a single nucleotide missense mutation, c.3659G>A (p.Gly1220Asp) (NM_033380.3), in exon 41 of the *COL4A5* gene. The mutant *COL4A5* had a change from guanine (G) to adenine (A) at base 3,706, which resulted in amino acid 1,220 of the encoded protein being changed from glycine (Gly) to aspartic acid (Asp). The *COL4A5* gene encodes the α5 chain of type IV collagen, which combines with the α3 and α4 chains of type IV collagen to form a triple helix structure (15). The main feature of the primary structure of type IV collagen was the repeated sequence of amino acids, which was represented by a glycine every three residues, and the polypeptide sequence was: -Gly-Xaa-Yaa-Gly-Xaa-Yaa-Gly-Xaa-Yaa-. Glycine is the amino acid with the lowest molecular weight of the amino acid composition of collagen. It has a single hydrogen atom in the side chain, has a larger side chain space, and allows the widest angle for the polypeptide chain to turn compared to other amino acids (16). The glycine in the Gly-Xaa-Yaa sequence is critical for forming collagen's triple helix structure. Substitution of glycine by other amino acids introduces steric hindrance and destabilizes the helical conformation, possibly leading to bulges or kinks in the linear collagen molecule. The type IV collagen α5 chain is the essential structural component of the basement membrane in the human GBM, cochlea, and lens (17). In this study, the glycine at position 1,220 was replaced by aspartic acid, which resulted in the interruption of the Gly-Xaa-Yaa repeat sequence and may have caused a weakened GBM and increased fragility of the podocyte cytoskeleton (16). Defective expression of GBM and abnormal stromal-podocyte interactions induce AS, with auditory and visual problems. The p.Gly1220Asp mutation of the *COL4A5* gene, which emerged in this study, has been listed as possibly pathogenic/pathogenic in the ClinVar database (https://www.ncbi.nlm.nih.gov/clinvar/) (variant identifier: 24666).

In our study, the wife was heterozygous, and her son was hemizygous for the *COL4A5* mutation, as the *COL4A5* gene was located on Xq22. The female mutation carriers in this family only showed isolated microscopic hematuria. However, the male family members who carried the mutation had more severe renal phenotypes, such as hematuria, proteinuria, renal dysfunction, and renal pathological changes. It was clear that there were significant differences in the course and severity of the disease between male and female patients. Female patients that are heterozygous for a pathogenic mutation have historically been defined as healthy carriers. However, subsequent studies have shown that female carriers display a spectrum of phenotypes that range from microscopic hematuria to ESRD and extrarenal manifestations. Approximately one in four female carriers will develop ESRD by age 60, and the risk increases with age. The mouse experiments of Rheault et al. (18) and the clinical case reports of Mastrangelo et al. (19) suggested that the extensive phenotypic characteristics of female heterozygotes may be related to digenic inheritance and skewed inactivation of the X chromosome. However, Günthner et al. (20) explored X inactivation in urine-derived cells of individual XLAS women. They found that X inactivation was not associated with age at first presentation, proteinuria, or glomerular filtration rate (eGFR). They found that the degree of X inactivation correlated with age, which may be an escape mechanism to avoid the expression of *COL4A5* mutations as individuals age. Male hemizygous carriers have more obvious genotype-phenotype correlations and more severe phenotypic features than female heterozygous carriers. Nearly 90% of men with XLAS develop early chronic kidney disease (CKD) and end-stage kidney disease (ESKD) by age 40. The study found that the type of mutation was an important factor that affects the severity of XLAS in men (6, 7). Male patients with truncating mutations develop ESKD at approximately 20 years old, while those with non-truncating mutations develop ESKD at 40. It is worth noting that XLAS, caused by *COL4A5* mutations, has considerable phenotypic heterogeneity and a combination of phenotypes (21–24). It may not even show typical clinical phenotypes (such as hematuria, proteinuria, and hearing and vision problems) or AS-like pathological changes, which is not conducive to early diagnosis and effective intervention (11). Therefore, in young patients with unexplained glomerular diseases, without typical manifestations, clinicians also need to consider *COL4A5* mutations. Genetic testing, based on next-generation sequencing, is crucial for the diagnosis of XLAS, which is a disease with high phenotypic and genetic heterogeneity (21–23, 25).

The traditional prenatal diagnosis technology, based on amniocentesis, has been the major means to prevent the birth of children with monogenic genetic diseases in the past. However, it is traumatic and carries a risk of miscarriage. After prenatal diagnosis confirms that the fetus has AS, the pregnant woman needs to terminate the pregnancy, which causes physical and psychological harm to her and her family. Using PGT-M, the blastocyst trophoblast cells can be taken for genetic analysis, and normal embryos can be selected for transplantation into the mother's uterus. That prevents the intergenerational transmission of single-gene diseases and prevents couples from deciding to terminate the pregnancy or having affected children. Currently, single-cell expansion methods suitable for PGT include MDA and MALBAC. MDA can give higher cell concentrations after amplification, but the amplification uniformity and coverage of MALBAC are better than MDA (26). The initial concentration of DNA used for WGA in PGT is low, and the exponential amplification may cause ADO. Allele dropout refers to the failure to amplify one allele in heterozygotes, which results in the site being falsely detected as homozygous. Allele dropout is one of the characteristics of single-cell WGA and is also a key factor that leads to the underreporting of single-base mutations (26). In this case, ADO may have resulted in a false negative call. Compared with MDA, the false positive and ADO rates of MALBAC are lower (27).

In summary, MALBAC had high amplification uniformity and a low ADO rate. The WGA product from MALBAC was applied directly to CNV-seq sequencing library preparation, and it had good results in CNV analysis and SNP detection. Therefore, we used MALBAC for gene amplification of trophoblast cells. While other reports describe the application of PGT-M in XLAS (28, 29), this is the first use of the MALBAC technique for WGA. In order to reduce the impact of ADO, we used polymorphic markers from the proband and his parents and linkage analysis of mutant genes to establish haplotypes. We found that the analysis of SNP markers by NGS was beneficial in ensuring the accuracy of PGT-M (30).

Through CNV analysis, SNP haplotype analysis, and Sanger sequencing, we determined that embryos E1, E2, and E4 did not carry any pathogenic mutations or chromosomal abnormalities. We combined the results of embryo morphology and PGT and used embryo E1 (4AA) for transplantation. The pregnancy was successful. Although we performed some work to avoid misdiagnosis during PGT, we could not altogether avoid the influence of chromosomal rearrangement, mosaicism, and ADO on the diagnostic results. It was still necessary to prove the results of PGT by amniocentesis in the second trimester. In this case, the results of genetic testing on amniotic fluid cells were consistent with the results of PGT-M, and a healthy baby boy was born.

In conclusion, we reported a family with XLAS caused by a *COL4A5* mutation. The family underwent PGT-M to prevent the transmission of the *COL4A5* pathogenic mutation to their child. Through MALBAC-based WGA, NGS-based haplotype analysis and prenatal diagnosis in the second trimester, to reduce the risk of misdiagnosis from PGT-M, the couple finally gave birth to a healthy baby boy. Our results confirmed the feasibility of PGT-M to block the intergenerational transmission of the *COL4A5* mutation, which has an important clinical application in the prevention and control of birth defects.

## Data Availability

The raw datasets analysed during the current study are not deposited in publicly available repositories because of considerations about the security of human genetic resources and patient anonymity. Requests to access these datasets should be directed to NL, liuneng10@gmail.com.
